# The Effect of Ag Nanoparticles and Multimicrobial Preparation as Factors Stabilizing the Microbiological Homeostasis of Feed Tables for *Cornu aspersum* (Müller) Snails on Snail Growth and Quality Parameters of Carcasses and Shells

**DOI:** 10.3390/ani10122260

**Published:** 2020-12-01

**Authors:** Andrzej Łozicki, Tomasz Niemiec, Robert Pietrasik, Sylwester Pawęta, Anna Rygało-Galewska, Klara Zglińska

**Affiliations:** 1 Division of Animal Nutrition, Institute of Animal Sciences, Warsaw University of Life Sciences, Ciszewskiego 8, 02-786 Warsaw, Poland; tomasz_niemiec@sggw.edu.pl (T.N.); anna_rygalo-galewska@sggw.edu.pl (A.R.-G.); klara_zglinska@sggw.edu.pl (K.Z.); 2 Institute of Materials Science and Engineering, Lodz University of Technology, Stefanowskiego 1/15, 90-924 Lodz, Poland; robert.pietrasik@p.lodz.pl (R.P.); sylwester.paweta@p.lodz.pl (S.P.)

**Keywords:** snail, *Cornu aspersum* (Müller), Ag nanoparticles, effective microorganisms (EM), feed tables

## Abstract

**Simple Summary:**

The farming of snails, unlike that of large farm animals, requires less space and financial resources, and snails are not as demanding. In field husbandry conditions, snails have access to green forage and are given concentrated mixtures on feed tables. In this maintenance system, it is important to carry out treatments stabilizing the microbiological balance of feed tables, where snail feces and feed refusals accumulate. This study analyzed the effect of paint with silver nanoparticles (nano-Ag) or a multimicrobial preparation applied to feed tables on the microbiological composition of the feed table environment, the growth and mortality of snails, and parameters assessing the quality of carcasses and snail shells. Results showed that the use of nano-Ag paint reduced the growth of bacteria, while the multimicrobial preparation reduced mold and yeast. Spraying feed tables with the multimicrobial preparation had a better effect on the growth of snails, while the use of nano-Ag paint reduced the mortality of the animals. The factors used did not have a negative effect on the quality of shells. The snails that had contact with nano-Ag paint showed a higher content of Ag in the carcasses and a greater degree of lipid peroxidation.

**Abstract:**

The aim of this research was to evaluate the effect of Ag nanoparticles (nano-Ag) used in the paint covering feed tables or a multimicrobial preparation applied to feed tables on the microbiological composition of the feed table environment, the growth and mortality of snails, and selected parameters for assessing the quality of carcasses and snail shells. The research was carried out in a farm of *Cornu aspersum* (Müller) snails. In the control (K) group, paint without nano-Ag was used. In two other groups (N-Ag and N-Ag + effective microorganisms (EM)), the feed tables were covered with the same paint as in the control group but with the addition of 100 mg/L of nano-Ag it (N-Ag group). Additionally, multimicrobial preparation (EM Bokashi^®^) at a concentration of 10% was spread on the tables in the N-Ag + EM group. In the last group (EM), the feed tables were covered with paint without nano-Ag, and only multimicrobial preparation was applied at a concentration of 10%. During the tests, the body weight of snails was measured three times, and swab samples were taken from the feed tables for the examination of microbiological composition. At the end of the experiment, the snails were killed, and the weight of the carcass and the size of the shell were measured. The content of Ag and the degree of lipid oxidation (thiobarbituric acid reactive substances (TBARS)) in the carcasses were analyzed, and the content of Ca and the crushing strength of the shells were determined. In the N-Ag and N-Ag + EM groups, a significant reduction in the total number of bacteria, fecal streptococci, and *Escherichia coli* was found, while there was also a reduction in mold and fungi in the N-Ag + EM and EM groups. In the K and EM groups, the mortality of animals was higher than in the nano-Ag groups. In subsequent weight checks, the highest body weight was found in the EM group and the lowest in the N-Ag and N-Ag + EM groups. In addition, the carcass weight and shell size in the N-Ag group was significantly lower compared to the K and EM groups. In the N-Ag and N-Ag + EM groups, a higher Ag content in the carcasses and a greater degree of lipid peroxidation were found. The Ca content of the shells was the highest in the N-Ag group, and the hardness of shells was the highest in the N-Ag and N-Ag + EM groups.

## 1. Introduction

Husbandry of large livestock, such as cattle, pigs, and poultry, is expensive. It is also connected with high water consumption and the emission of a significant amount of pollutants to the environment, such as nitrogen compounds and greenhouse gases. This situation favors the development of alternative and sustainable systems, the implementation of innovative and safe technologies, and the production of animal protein from invertebrates, including mollusks.

Unlike large farm animals, snails require less space, are not as demanding, and do not require large financial investments to start farming.

Snail meat, which is rich in nutrients and easily digestible, can be an alternative to beef, pork, and poultry. The dietary properties of snail meat are determined by the high biological value of its protein content, which is the source of essential amino acids, as well as the low fat content, with snail meat containing as much as 75% unsaturated fatty acids [1,2]. The meat of these mollusks is also a source of minerals (100 g of fresh snail meat covers about 60% of the daily requirement for calcium and 25% of the daily requirement for iron in an adult human) as well as vitamins A, B6, B12, K, and folic acid [3,4,5,6].

In field husbandry conditions, snails have access to green fodder and are given concentrated feed on feed tables. In this maintenance system, it is important to carry out treatments stabilizing the microbiological balance of feed tables, where snail feces and feed refusals accumulate. At high temperatures and humidity, the growth of putrefying bacteria and fungi is favored. This may have a negative impact on the health of snails and consequently their mortality, growth, and quality of shells. One way to hygienize tables is to cover their surface with paint with the addition of Ag nanoparticles, which have strong antibacterial and antifungal properties [7,8,9,10]. These properties have been used for research on the introduction of nano-Ag in food preservation packaging [11,12,13] and have already found practical application for similar solutions [14].

Another method of hygienization is the application of preparations containing live microorganisms to the feed tables, e.g., preparations of effective microorganisms (EM) Bokashi^®^. The technology of producing EM is based on a mixture of various yeast species, aerobic and anaerobic bacteria that produce organic acids, enzymes, compounds with antioxidant activity, and metal chelates [15]. Therefore, the use of EM in animal environments, or their addition to feed or water, can have a positive impact on the living environment of animals and lead to better growth, better feed conversion, and improved animal health [16,17,18,19].

The coating of feed tables with nano-Ag paint or EM preparation should reduce the growth of putrefying bacteria and fungi. This improvement of hygienic conditions results in better growth of animals and the quality of shells and carcasses. The aim of this research was to assess the impact of nano-Ag used in the paint covering feed tables or EM Bokashi^®^ preparation applied to feed tables on the microbiological composition of the feed table environment, the growth and mortality of snails, and selected parameters for carcass and shell quality assessment.

## 2. Materials and Methods

### 2.1. Animals and Experimental Design

**Figure 1:**
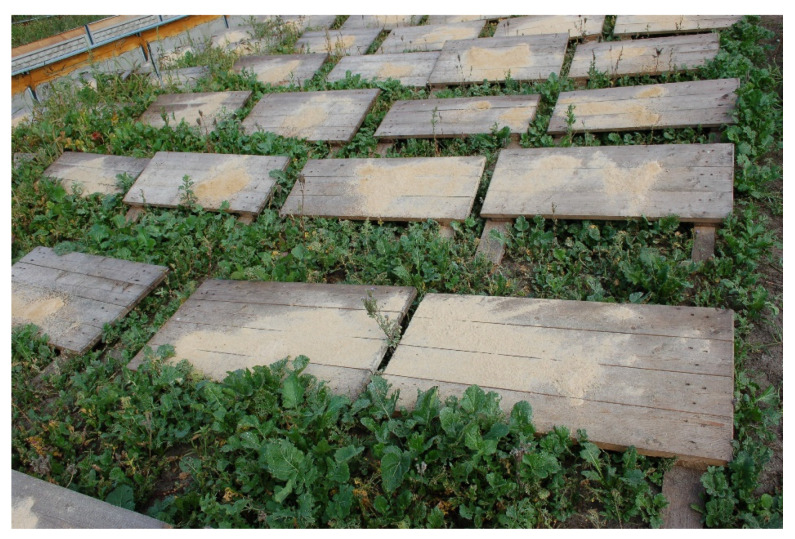
Feed tables on the experimental plot.

The research was carried out in a snail farm during their fattening period from June to September 2018. The subject of research was the European subspecies of brown snail *Cornu aspersum* (Müller), which is popular among consumers and suitable for intensive production. During the research, four experimental variants and two replicates/experimental plots were used in each variant. The snails were introduced to the experimental plots at the beginning of June. The area of the individual plots was 625 m^2^, with an average number of 350 snails per m^2^. In each plot, there were 400 feed tables, each with an area of 0.6 m^2^, made of beech boards (Figure 1). The surface of the tables was covered with a paint intended for covering surfaces in contact with food, namely, a solvent-free epoxy system.

In the variant/control group, the prepared feed tables were covered with paint without the addition of nano-Ag (K group). In two subsequent groups/experimental variants (N-Ag and N-Ag + EM groups), the feed tables were covered with the same paint as in the control group but with the addition of 100 mg/L of nano-Ag (N-Ag group). Additionally, in one variant, EM Bokashi^®^ preparation with a concentration of 10% (N-Ag + EM group) was spread on the feed table. In the last variant/experimental group, the feed tables were covered with paint without the addition of nano-Ag, and only the EM Bokashi^®^ preparation was spread on it at a concentration of 10% (EM group). The paint with nano-Ag was obtained by adding nanosilver “Al 2100” (silver nanoparticle 30–60 nm, polyvinyl alcohol (PVA) coating, alcohols suspensions, 1000 ppm, Amepox, Łódź, Poland) to the solvent-free epoxy system in a ratio of 1:10 (*v*/*v*).

The EM Bokashi^®^ preparation used in the experiment is manufactured by the commercial company Greenland Technologia EM, Janowiec, Poland, and contains a mixture of microorganisms, as described by Laskowska et al. [20], namely, *Sacharomyces cerevisiae* (Y200007) 5 × 104 CFU/g, *Lactobacillus casei* (ATCC 7469) 5 × 108 CFU/g, *Lactobacillus plantarum* (ATCC 8014) 5 × 108 CFU/g, *Enterococcus faecalis* (UC-100 (CGMCC No.1.0130)) 2.5 × 106 CFU/g, *Enterococcus faecium* (NCIMB SF68) 5 × 109 CFU/g, *Bifidobacterium bifidum* (ATCC 29521) 5 × 108 CFU/g, *Bifidobacterium pseudolongum* (ATCC 25526) 5 × 108 CFU/g, *Bacillus licheniformis* (DSM 5749) 4 × 109 CFU/g, *Bacillus cereus var. toyoi* (NCIMB 40112) 4 × 109 CFU/g, *Bacillus subtilis* (MA139) 4 × 1011 CFU/g, and *Clostridium butyricum* (MIYAIRI 588 (CBM588)) 1 × 108 CFU/g.

The EM Bokashi^®^ preparation in the form of an aqueous suspension was spread on the surface of the feed tables and under the tables using a hand-held sprayer. During the entire study period (June to September 2018), the preparation was used once a week on Mondays at a fixed time of 8–10 a.m.

The experiment scheme is presented in Table 1.

In all the plots, snails had access to Brassica rapa var. sylvestris fodder and were fed a concentrate mixture that included corn, wheat, extracted soybean meal, corn dried distillers’ grains with solubles (DDGS), fodder yeast, oil, fodder chalk, 1-calcium phosphate, NaCl, and premix. The nutritional value of 1 kg of the mixture were as follows: total protein, 170 g; lysine, 9 g; met + cys, 4.8 g; crude fiber, 33 g; crude fat, 50 g; Ca, 130 g; and P, 7.5 g. The animals had constant access to concentrated feed. It was given twice a day at 9–10 a.m. and 5 p.m. Green fodder samples were collected once a month, and its basic composition was analyzed. On average, for the entire period of the experiment, the basic composition of green fodder was as follows: dry weight, 164 g; total protein, 191 g/kg dry matter (DM); crude fiber, 186 g/kg DM; neutral detergent fiber (NDF), 394 g/kg DM; raw fat, 42 g/kg DM; raw ash, 134 g/kg DM; Ca, 26 g/kg DM; and P, 7.2 g/kg DM.

The remaining leftovers and snail droppings were removed from the tables before the fresh feed was distributed.

In all the plots, as a standard procedure used on the farm, an automatic field sprinkler system was installed, ensuring a relative humidity of 85–95% between 9 p.m. and 3 a.m.

### 2.2. Experimental and Analytical Procedures

Two-day-old snails were introduced to the experimental plots at the beginning of June (5 June). During the study, the body weight of snails was measured three times at intervals of about one month: measurement I at the beginning of July (8 July), measurement II at the beginning of August (5 August), and measurement III at beginning of September (5 September). The body weight of the snails was measured according to the following procedure: 8 random samples were weighed in each experimental plot, which consisted of 10 snails. The places where the snails were collected (1 sample weighted = 10 snails) were randomly marked on the diagonal of the plots.

Similar to measurements of the body weight of snails, the microbiological analysis of the feed tables was also performed three times: beginning of July (8 July), beginning of August (5 August), and beginning of September (5 September). Prior to collecting microbial samples, the surfaces were washed with deionized water and dried. The prepared sterile template in the form of a plastic frame with an area of 100 cm^2^ was placed on the tested surface. For each sampling time, a disposable sterile swab (BD CultureSwab™ Cary Blair agar) was wetted with sterile distilled water, and the same rubbing cycle was then performed on the surface along lines parallel to the long side of the frame from top to bottom every 1 cm. After the swabs were collected, the swab was placed in the transport medium. The samples were placed in a refrigerated container at 4 °C and transported to the laboratory within 4 h.

At the end of the study, during the harvest of the snails prior to sale, 30 snails were collected from each test plot and then killed by chilling and freezing them. The carcass was removed from each snail, separating it from the shell. The content of Ag and the degree of lipid oxidation (thiobarbituric acid reactive substances (TBARS)) in the carcasses were determined. In the case of shells, their Ca content and crushing strength were determined.

Snail mortality for the study period was calculated as the difference between the average number of animals released into individual plots and the estimated number of animals collected from individual plots. The number of collected snails was calculated by dividing the total weight of snails collected from a given plot by the average body weight of one snail from a given experimental plot.

The chemical composition of the feeds was determined according to AOAC [21]: moisture and dry matter by drying at 105 °C to constant weight, crude ash by incineration at 550 °C for 6 h, crude protein (N × 6.25) using the micro-Kjeldahl technique (Kjeltec System 1026 Distilling Unit, Foss Tecator, Sweden), and crude fat after extraction with petroleum ether by the Soxhlet method.

The NDF in feed was determined according to the method by Van Soest et al. [22]. The NDF was expressed as the ash-free residue after extraction with boiling neutral solutions of sodium lauryl sulfate and ethylenediaminetetraacetic acid (EDTA) in a Tecator apparatus.

For microbial analysis of the surface of feed tables, serial 10-fold dilutions in saline solution (up to 10^−5^) of bacterial suspensions were prepared. Then, 100 µL of each dilution was spread on Petri dishes containing the diagnostic medium (performed in triplicate). After incubation, in conditions required for selected groups of microorganisms, the number of colonies formed in the Petri dishes was counted. The analysis was performed according to the following standards: fecal enterococci (1 CFU/swab), PN-EN ISO 7899-2: 2004; yeasts and molds (1 CFU/swab), PN-ISO 21527-1: 2009; *Escherichia coli* B-glucuronidase positive (1 CFU/swab), PN-ISO 16649-2: 2004; *Listeria* monocytogenes, PN-EN ISO 11290-1: 1999/A1: 2005; and total number of microorganisms at 30 °C (1 CFU/swab/g), PN-EN ISO 4833: 2004.

Mineral components were determined by inductively coupled plasma optical emission spectrometry (ICP-OES) after microwave-assisted acid digestion. Test samples were dried at the temperature of 60 °C and then ground. The material prepared in this way was mineralized in concentrated HNO_3_ using microwave mineralization. In the mineralized material, the minerals (Ca, P, and Ag) were determined with an ICP-OEC equipment. The samples were analyzed in duplicate, and each sample was measured in triplicate.

The level of malondialdehyde, the main product of lipid oxidation, reacting with TBA was determined according to the methodology presented by Uchiyam and Mihar [23]. The absorbance at 532 nm was measured using a Tecan Infinite M200 spectrophotometer. The results are presented as concentrations in samples of compounds that react with TBARS. The test tissue was homogenized in 1% potassium chloride solution and then centrifuged at 2000 rpm for 15 min at 4 °C. Then, 1% phosphoric acid, 1% potassium chloride, 2% butylhydroxyanisole (BHA), and 0.4% TBA were added to the resulting filtrate. After adding the reagents and mixing, the sample was capped and placed in a water bath at 100 °C for 60 min. After cooling, 4 mL of butanol was added to the sample and shaken for 2 min. Absorbance of compounds dissolved in the butanol phase was read at 532 nm. The results were read in nmol/mL from a standard curve made against 1,2,3,3 tetraethoxypropane (TEP).

The shell strength was measured using a Zwick 1120 testing machine (Z 5.0 Zwicki–Line, Ulm, Germany) with a warhead equipped with a Warner–Bratzler blade with a preforce setting of 0.2 N. The blade movement speed was 5 mm/min.

### 2.3. Statistical Analysis

Results obtained were developed statistically using one-way analysis of variance (ANOVA) with Statgraphics 6.0 Plus software. Significant differences between groups were identified using the *F*-test. Results are presented in tables as mean values of parameters, standard errors of the means, and statistical significance of the effect.

## 3. Results

### 3.1. Microbial Community on the Surface of the Feed Tables

Table 2 shows the mean measurements of the microbiological community of swabs collected from the surface of the feed tables in the studied experimental groups. The lowest total number of bacteria was found on feed tables covered with nano-Ag paint, i.e., the N-Ag and N-Ag + EM groups, and it was significantly less compared to the control (K) group (*p* ≤ 0.01) and also compared to the EM group (*p* ≤ 0.01) for the N-Ag group. Significant differences in the total number of bacteria on the feed tables were also found between the groups with nano-Ag, with the N-Ag group having significantly less bacteria compared to the N-Ag + EM group (*p* ≤ 0.05). In the N-Ag and N-Ag + EM groups and in the EM group, there were significantly fewer fecal streptococci (*p* ≤ 0.01) compared to the control (K) group. The presence of these bacteria on the tables of the N-Ag group was also significantly less compared to the EM group (*p* ≤ 0.05). Similar relationships were found for *E. coli*, with the presence of these bacteria being significantly less in the N-Ag and N-Ag + EM groups compared to the K group (*p* ≤ 0.01) and significantly less in the N-Ag group compared to the EM group (*p* ≤ 0.05).

Comparing the total number of bacteria and *E. coli* between the K and EM groups, the differences were not significant, although they were both lower in the EM group. However, in the case of fecal streptococci, there was significantly less of these bacteria in the EM group compared to the K group (*p* ≤ 0.05). *Listeria* monocytogenes were not found on the fodder tables in any of the tested variants.

The number of mold and yeast colonies was significantly lower in the N-Ag + EM and EM groups compared to the K and N-Ag groups (*p* ≤ 0.05).

### 3.2. Body Weight Gain and Mortality of Snails

During the first measurement (I), the snails from the control (K) and EM groups had the highest body weight (Table 3). The weight of the K group snails was significantly higher compared to the N-Ag and N-Ag + EM groups (*p* ≤ 0.01) as well as the EM group (*p* ≤ 0.05). The snails from the EM group weighed significantly more compared to the N-Ag + EM (*p* ≤ 0.01) and N-Ag (*p* ≤ 0.05) groups. In the second measurement (II), the EM group had the highest body weight, and it was significantly higher than in the other groups (*p* ≤ 0.01). The body weight of snails from the K group was also significantly higher than in the N-Ag group (*p* ≤ 0.01). The relationships in the third measurement (III) were similar. The EM group of snails had the highest body weight, significantly higher than the others (N-Ag and N-Ag + EM, *p* ≤ 0.01, K, *p* ≤ 0.05), and the body weight of the K group snails was higher than the N-Ag (*p* ≤ 0.01) and N-Ag + EM (*p* ≤ 0.05) groups.

**Figure 2:**
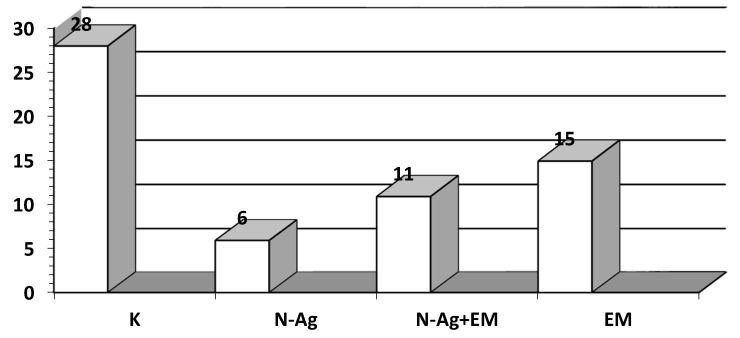
Estimated percentage (%) mortality of *Cornu aspersum* (Müller) snails for the fattening period on the experimental plots. EM: effective microorganisms.

The results determining the mortality of snails were not statistically analyzed. However, the estimated losses, presented in Figure 2, indicate that the mortality rate compared to the control (K) group was more than two times lower in the N-Ag + EM group and more than four times lower in the N-Ag group. A lower mortality compared to the control (K) group was also visible in the EM group. However, it was higher compared to the N-Ag and N-Ag + EM groups.

### 3.3. Carcass Weight and Evaluation of Shells

The highest weight of a single carcass was obtained from snails from the EM and K groups, and they were significantly higher than the N-Ag group (*p* ≤ 0.05) (Table 4). This is due to the highest final body weight of snails in the EM and K groups (Table 3). In the case of the parameters determining the size of the shell, significant differences were found in the length of the shell, which was the smallest in the N-Ag group (*p* ≤ 0.05). The smaller width of the shells was also visible in the N-Ag group, but the difference in relation to the other groups was not statistically significant.

The highest Ca content was found in the shells in the N-Ag group, and it was significantly higher than in the other groups (*p* ≤ 0.01) (Table 5). Compared to the control (K) and N-Ag + EM groups, the content of Ca in the shells of the EM group was also higher (*p* ≤ 0.01). The consequence of the highest content of Ca in the shells of snails from the N-Ag group was the highest crushing strength in this group, measured by the crushing force of the shells, which was significantly higher than in the K and EM groups (*p* ≤ 0.01). In the N-Ag + EM group, the compressive force of the shells was similar to the N-Ag group and significantly higher than in the K and EM groups (*p* ≤ 0.05).

### 3.4. Ag Content and Oxidation State of Snail

A significant influence of the nano-Ag paint covering the feed tables on the Ag content in the body of snails was found (Table 6). In both the N-Ag and N-Ag + EM groups, the Ag content in the carcasses was significantly higher compared to the K and EM groups (*p* ≤ 0.01).

In the carcasses of snails from the N-Ag and N-Ag + EM groups, a significantly higher concentration of TBARS was also found compared to the K and the EM groups (*p* ≤ 0.01). Within the groups with the application of nano-Ag, a significantly higher TBARS concentration was found in the N-Ag group compared to the N-Ag + EM group (*p* ≤ 0.05).

## 4. Discussion

The results of the research indicate a direct effect of the surfaces covered with antimicrobial layers on inhibiting the growth of bacteria, including potential pathogens and mold. A strong influence of the surfaces covered with nano-Ag on the limitation of bacterial growth and no effect of this factor on the development of fungi was found. In the case of using the preparation with effective microorganisms, there was also a reduction in the quantity of bacteria on the feed tables but not as visible as with the use of nano-Ag. The use of the EM preparation, on the other hand, significantly reduced the development of mold and yeast, which did not occur when using nano-Ag. Therefore, our research shows the effectiveness of using nano-Ag and effective microorganisms on feed tables to limit the growth of bacteria and the lack of effect of nano-Ag on limiting the growth of mold and yeast.

The antibacterial effectiveness of nano-Ag in the paint might result from the migration of nanosilver to the environment and its interaction with the microorganisms living there. The migration mechanism occurs when nano-Ag is used in food preservation packaging as well [13]. The effectiveness of the antibacterial activity of silver has been confirmed in many studies [7,8,9,10]. The mechanism of the antibacterial action of silver nanoparticles may result from the capture of the released silver ions by the bacterial cell and then disruption of ATP production and DNA replication. It has also been indicated that nanoparticles and silver ions induce the production of reactive oxygen species (ROS) or may cause direct damage to cell membranes and influence the modulation of cell signaling [9,24,25].

The obtained results may indicate a negative effect of nano-Ag used in the paint covering the feed tables on the growth and development of snails. McShan et al. [26], based on a review of the literature, indicated that the toxicity of nano-Ag may result from its impact on proteins, nucleic acids, and the cell membrane or the initiation of oxidation processes. However, they did not indicate which of these mechanisms is most responsible for the toxicity of nanosilver.

In the present research, the analysis of the lipid oxidation state as an indicator of the oxidative state of organisms showed the highest concentration of TBARS in the groups in which nano-Ag was applied (Table 6). This indicates a greater intensity of oxidation processes in these groups. It may therefore be a factor that adversely affects the growth and development of snails. The highest final body weight, as well as that during the second measurement, was found in the EM group, i.e., without the addition of nano-Ag in the paint but with the spraying of EM Bokashi^®^ preparation on the feed tables. Research has shown that this preparation may have probiotic effects for animals [19]. The studies by Charrier et al. [27] indicate that the use of rich mixtures in snail nutrition reduces the decomposition of cellulose in the gut. In our research, the contact of snails with the EM Bokashi^®^ preparation, which due to the participation of various groups of microorganisms in it, might have had an impact on the qualitative and quantitative composition of the gut microflora. This, in turn, might have resulted in better digestibility of the feed, including green fodder cellulose, and the effect of this was an improvement in the energy balance and consequently better growth. However, the spray effect of EM Bokashi^®^ was not visible when it was used together with the application of nano-Ag paint.

There are few studies in which probiotic preparations were used in the breeding of snails, added to feed or water, or introduced into the environment. Ligaszewski and Pol [28] used sprinkling greenhouse and earth snail pens with a probiotic preparation other than EM Bokashi^®^. However, this did not result in obtaining higher body weight of animals compared to the control group. On the other hand, there are numerous studies on poultry or pigs in which probiotic preparations were administered in feed or water or introduced into the environment. Many of these studies found improvements in animal performance [29,30], but there are also studies where positive effects of probiotics were not found [31,32]. With regard to studies using preparations with effective microorganisms (EM Bokashi^®^), da Cruz et al. [33] sprayed an aqueous solution of EM on a litter in the hovel and studied its impact on the environment of the hovel and the development of broiler chickens. However, they did not find a positive effect of the applied EM Bokashi^®^ preparation on the production results of chickens. Esatu et al. [34], on the other hand, administered EM to broiler chickens in the form of addition to water and feed and found positive effect on animal growth and feed consumption per kilogram of growth. Aly et al. [35] investigated the effect of EM or EM alone with different energy components introduced into the water on water quality and the development and use of feed by Nile tilapia (*Oreochromis niloticus*). The authors found a positive effect on tilapia growth with both EM supplementation and EM supplemented with carbohydrate feed.

The lowest mortality in the groups with the application of nano-Ag might have resulted from the clear reduction in the number of bacteria on the feed tables in these groups, which improved their hygienic conditions. In this way, snails, especially younger and more sensitive snails, were less exposed to pathogens. The use of only sprays with the EM preparation, although its positive effect on the feed table environment was noted, did not result in such a clear decrease in the mortality of snails as with the use of nano-Ag. This might indicate that limiting the development of potentially pathogenic bacteria in the habitat of snails (N-Ag and N-Ag + EM groups) rather than restricting the development of mold and yeast (EM group) serve to reduce the mortality of these animals. Ligaszewski and Pol [28] used sprinkling water solutions of preparations with garlic, probiotics, antibiotics, and their combinations in greenhouse and earth snail farms and found snail losses ranging from a few to even 50%. The lowest losses occurred with the use of the garlic preparation and the highest with the antibiotic preparation. When spraying with the probiotic preparation, the losses were smaller compared to the preparation with the antibiotic, but differences were not visible compared to the control group. Therefore, although it is difficult to compare the results of these authors with ours, a similar tendency is visible, namely, that introducing a probiotic preparation into the habitat of snails does not significantly reduce the loss of snails.

As a consequence of the higher final weight of snails in the EM and K groups, there was also a higher carcass weight compared to the N-Ag and N-Ag + EM groups.

When assessing the size of the snail shell in terms of its commercial value, despite the shorter length in the N-Ag group, it can be concluded that it was good in all groups and fell within the accepted standards. Lazaridou-Dimitriadou et al. [36] states that the commercial size of *Cornu aspersum* (Müller) snails should be 25–32 mm. In our study, in the group where snails were the smallest (N-Ag), their size was 30.31 mm, while the size even exceeded 33 mm in the K and EM groups. The sizes achieved by the nano-Ag groups can be considered to be more in line with commercial expectations.

The experimental factors applied did not have a negative effect on the Ca content of the shells, and even in the N-Ag and EM groups, it was significantly higher compared to the control group. Thus, this research shows that the applied experimental factors do not have a negative effect on the quality of the shells, which is important for breeders from the point of view of the commercial value of the product, and even the use of nano-Ag can improve the quality of the shells.

The significantly higher silver content in the carcasses in the nano-Ag groups indicates the likely release of silver ions/nanoparticles from the paint surface and their absorption by animals staying on the feed tables. Although the difference in the concentration of silver between the animals living in the environment with nano-Ag and the groups free from these nanomaterials was twofold, the concentration of silver in animal tissues observed in this experiment, based on European Food Safety Authority (EFSA) data [37], can be considered as nontoxic to humans.

The analysis of snail carcasses in terms of the content of fat oxidation products (TBARS), however, indicates the pro-oxidative properties of silver nanoparticles contained in the paint covering the feed tables. Higher Ag content in the carcasses of snails from the N-Ag and N-Ag + EM groups was also accompanied by a higher concentration of TBARS in these groups, which indicates more intense fat oxidation processes. The lower level of lipid oxidation in the N-Ag + EM group compared to N-Ag group may also indicate the alleviating effect of using the probiotic preparation with EM. This could be due to improvement of the microbiological composition of the gastrointestinal tract of snails in the N-Ag + EM group and thus better use of dietary ingredients, including bioactive compounds, which had an antioxidant effect. However, the use of the EM preparation did not fully neutralize the pro-oxidative effect of nano-Ag as the TBARS level in the N-Ag + EM group was higher compared to the control (K) and EM groups.

The issue of oxidative properties of silver nanoparticles has been analyzed in many studies. Docea et al. [38] found that silver nanoparticles, when administered to rats in the form of various compounds, had an antioxidant activity, including a reduction in the level of TBARS in the plasma compared to the control group, which was not administered nano-Ag compounds. The antioxidant effect of silver nanoparticles was also found in the studies by Singh et al. [39] and Das et al. [40]. It has been indicated that these properties of silver nanoparticles allow their use in products used in medicine [41]. However, in studies by Patlolla et al. [42], administration of silver nanoparticles to rats increased the formation of ROS. In studies by Foldbjerg et al. [43], silver nanoparticles induced oxidation processes on human monocyte lines, leading to their apoptosis and necrosis. The mechanism of oxidative stress induction by silver nanoparticles may be related to the generation of reactive oxygen species (ROS) inside cells by silver ions on the surface of nanoparticles or released from them [43,44,45].

The increased intensity of oxidation processes found in our research in the nano-Ag groups could be the cause of worse growth of snails in these groups.

## 5. Conclusions

The use of nano-Ag paint on feed tables significantly reduced the growth of bacteria but did not affect the growth of fungi. However, the use of EM preparation greatly reduced the number of molds and yeasts. Spraying feed tables with only the EM preparation had a positive effect on the growth of snails and their final weight, while the use of nano-Ag paint resulted in deterioration of their growth, lower final weight, and consequently lower carcass weight. However, the use of nano-Ag limited the mortality of the animals. The experimental factors used did not have a negative effect on the quality of the carcass. The snails that had contact with the nano-Ag paint showed a higher content of Ag in the carcasses and a greater degree of lipid peroxidation. The preparations used in this research can be used in the husbandry of snails.

## Figures and Tables

**Table 1:** Scheme of experimental groups.

Specification	Experimental Groups			
	K	N-Ag	N-Ag + EM	EM (Effective Microorganisms)
Variants of experiment	Paint-coated feed tables without the addition of nano-Ag	Feed tables covered with paint with the addition of nano-Ag	Paint-coated feed tables with nano-Ag + EM 10%	Paint-coated feed tables without the addition of nano-Ag + EM 10%
The number of plots in individual variants	2	2	2	2
Number of feed tables per plot	400	400	400	400

**Table 2:** Means of measurements of microbiological community of swabs taken from the surface of feed tables in the control and experimental groups.

Item	Experimental Groups				SEM	*p*-Value
	K	N-Ag	N-Ag + EM	EM		
Total bacterial count (CFU/swab)	3.3 × 10^6 BD^	1.5 × 10 ^6Aa^	2.2 × 10^6 Cb^	2.5 × 10^6 B^	2.22 × 10^5^	0.001
Fecal streptococci (CFU/swab)	5.0 × 10^2 B^	1.9 × 10^2 Aa^	2.6 × 10^2 A^	2.8 × 10^2 Ab^	38.30	0.000
*Listeria* monocytogenes (CFU/swab)	absent	absent	absent	absent	-	-
*Escherichia coli* (CFU/swab)	3.4 × 10^4 B^	2.1 × 10^4 Aa^	2.4 × 10^4 A^	2.9 × 10^4 b^	2.03 × 10^3^	0.001
Mold and yeast (CFU/swab)	7.2 × 10^5 B^	7.5 × 10^5 B^	1.53 × 10^5 A^	2.0 × 10^5 A^	3.65 × 10^4^	0.000

Values in the same row, appearing in pairs, differ at AB, CD: *p* ≤ 0.01; ab: *p* ≤ 0.05; EM: effective microorganisms; SEM: standard error of means.

**Table 3:** Average body weight (g) of 10 *Cornu aspersum* (Müller) snails in the control and experimental groups in subsequent measurements during fattening.

Item	Experimental Groups				SEM	*p*-Value
	K	N-Ag	N-Ag+ EM	EM		
I	21.72 ^Aa^	17.54 ^Bd^	15.90 ^BD^	19.54 ^Cbc^	0.687	0.000
II	96.52 ^BC^	85.25 ^BD^	90.94 ^B^	106.44 ^A^	2.424	0.000
III	104.46 ^Cbc^	94.23 ^BD^	96.90 ^Bd^	113.96 ^Aa^	2.611	0.000

Values in the same row, appearing in pairs, differ at AB, CD: *p* ≤ 0.01; ab, cd: *p* ≤ 0.05; EM: effective microorganisms; SEM: standard error of means.

**Table 4:** Carcass weight and shell size of *Cornu aspersum* (Müller) snails in the control and experimental groups.

Item	Experimental Groups				SEM	*p*-Value
	K	N-Ag	N-Ag + EM	EM		
Carcass weight (g)	6.95 ^a^	5.54 ^b^	6.76 ^ab^	7.40 ^a^	0.461	0.045
Shell length (mm)	33.42 ^a^	30.31 ^b^	32.04 ^a^	33.77 ^a^	0.772	0.012
Shell width (mm)	23.49	22.48	23.03	24.48	0.725	0.556

Values in the same row, appearing in pairs, differ at ab: *p* ≤ 0.05; SEM: standard error of means.

**Table 5:** The share of calcium and hardness of *Cornu aspersum* (Müller) snail shells in the control and experimental groups.

Item	Experimental Groups				SEM	*p*-Value
	K	N-Ag	Ag + EM	EM		
Ca (g/kg)	387.0 ^BD^	417.0 ^A^	389.6 ^BD^	401.9 ^BC^	2.342	0.000
The crushing force of the shells (N)	115.2 ^B^	138.1 ^A^	135.9 ^A^	115.0 ^B^	4.086	0.001

Values in the same row, appearing in pairs, differ at AB, CD: *p* ≤ 0.01; EM: effective microorganisms; SEM: standard error of means.

**Table 6:** Silver content and the level of the oxidative state index (thiobarbituric acid reactive substances (TBARS)) in the carcasses of *Cornu aspersum* (Müller) snails in the control and experimental groups.

Item	Experimental Groups				SEM	*p*-Value
	K	N-Ag	N-Ag + EM	EM		
Ag (µg/kg)	14.87 ^B^	29.62 ^A^	31.7 ^A^	13.55 ^B^	0.927	0.000
TBARS (nmol/mg lyophilisate)	0.63 ^BD^	1.01 ^A^	0.86 ^BC^	0.68 ^BD^	0.023	0.000

Values in the same row, appearing in pairs, differ at AB, CD: *p* ≤ 0.01; EM: effective microorganisms; SEM: standard error of means.
